# Evaluation of aqueous chlorine and peracetic acid sanitizers to inactivate protozoa and bacteria of concern in agricultural water

**DOI:** 10.1128/aem.01653-24

**Published:** 2024-12-06

**Authors:** Kyle J. McCaughan, Zoe Scott, Channah Rock, Kalmia E. Kniel

**Affiliations:** 1 Department of Animal and Food Sciences, University of Delaware 5972 , Newark, Delaware, USA; 2 Department of Environmental Science, University of Arizona 165914 , Maricopa, Arizona, USA; Centers for Disease Control and Prevention, Atlanta, Georgia, USA

**Keywords:** agricultural water, chlorine, peracetic acid, *Cryptosporidium parvum*, *Cyclospora cayetenensis*, *Escherichia coli*, *Salmonella*, irrigation

## Abstract

Agricultural water is a potential source of microbial contamination whereby *Escherichia coli, Salmonella, Cryptosporidium,* and *Cyclospora cayetenensis* can enter the food supply. To reduce this risk, effective sanitization of agricultural water may be critical to food safety. As such, it is important to investigate the effects of aqueous peracetic acid (PAA) and chlorine (Cl) on bacteria and protozoa at different treatment times and temperatures in agricultural water with respect to key water characteristics. Multiple concentrations of each sanitizer, ranging from 3 to 200 ppm, were prepared in recently collected agricultural water, the solution was brought to the desired temperature, and the target organisms were added and left for the desired contact time (5 or 10 minutes) when sodium metabisulfite was added to neutralize the sanitizers. Bacterial samples were enumerated on MacConkey or XLT4 agar. Samples with protozoa were added to mammalian cell culture (HCT-8 cells for *Cryptosporidium parvum* and MDBK cells for *Eimeria tenella*). After 48 hours, the infected cells were collected, DNA extracted and infectivity assessed by quantitative PCR (qPCR). Low and high concentrations of sanitizer were effective at eliminating bacteria with Cl being significantly (*P* < 0.05) more effective. The greatest reductions in *E. coli* and *Salmonella* (3.48 log and 2.5 log cfu/mL, respectively) were observed after 10 minutes of exposure to 10 ppm Cl. Concentrations of sanitizer 50 ppm and lower resulted in insignificant (*P* > 0.05) reductions in parasite infectivity of less than 1 log for both organisms. A 200 ppm PAA treatment reduced infectious oocyst populations by 3.8 log for *C. parvum* and 2.6 log for *E. tenella*, with Cl being significantly (*P* < 0.05) less effective against these organisms.

**IMPORTANCE:**

This research is critical to inform decisions regarding the application and use of sanitizers in pre-harvest agricultural water settings to enhance food safety. Understanding the effectiveness of chlorine (Cl) and peracetic acid (PAA) on bacteria and protozoa will allow for the more efficient and practical use of these sanitizers, thus improving agricultural practices in ways that are beneficial to both growers and consumers.

## INTRODUCTION

Water is critical for a number of agricultural practices including livestock care, post-harvest processing of produce, and cleaning, however, in an agricultural setting the largest use of freshwater comes in the form of irrigation. In the United States, ground and surface water used for irrigation accounted for approximately 42% of the nation’s total freshwater withdraws in 2015, with ground and surface water making up 16% and 21%, respectively (1). However, the possibility of contamination of agricultural water by pathogens, including shigatoxigenic *Escherichia coli*, *Salmonella*, *Cryptosporidium parvum*, and *Cyclospora cayetenensis*, has raised concerns regarding food safety and the potential for outbreaks of foodborne illness (2). To address these concerns the use of sanitizers, such as peracetic acid (PAA) and chlorine (Cl), has increased in popularity in pre-harvest agricultural water, even though their efficacy in the challenging matrix that is surface water remains unclear. Understanding the relationship between agricultural water parameters and the reductions in pathogen populations will allow for a more pragmatic approach to the implementation of sanitizers in agricultural surface water, improving cost-efficiency for growers and producing safety for consumers.

Peracetic acid is a highly reactive compound, consisting of hydrogen peroxide and acetic acid. It demonstrates broad-spectrum antimicrobial activity while also being easily formulated and added to agricultural water systems. Furthermore, in water, peracetic acid degrades quickly into oxygen and carbon dioxide, meaning no harmful residues are introduced to the food surface or returned to the environment (3). When acting as a disinfecting agent peracetic acid oxidizes the membranes of microorganisms; when the membranes are oxidized electrons are able to rapidly flood the microbes causing them to lyse or otherwise be deactivated (4). While its quick activity and lack of residue make it an appealing choice for water sanitization, the effectiveness of peracetic acid may be significantly impacted by pH and temperature (4), therefore the relationship between these parameters must be researched and thoroughly considered when implementing this sanitizer in routine agricultural practices.

Chlorine has been a staple in water treatment for decades (5) due to its proven efficacy in pathogen deactivation and elimination. Chlorine eliminates microorganisms by breaking various chemical bonds within the organisms and inhibiting enzymatic activity. When chlorine interacts with hydrogen atoms within an organism the molecules are forced to change shape or fall apart, when this happens the microorganisms can no longer function (6). Like PAA, Cl is also easily formulated while having the added benefit of being pervasive and inexpensive, meaning it is easy to use in abundance. That being said, a downside of Cl for the sanitization of agricultural water is that it is highly reactive with organic material and its function relies heavily on the conditions of the water to which it is being added (7). When added to surface water that is highly turbid the active Cl will begin reacting with all biological matter, resulting in the Cl being rapidly “spent” and less effective at eliminating the organisms of interest (7).

The Food Safety Modernization Act (FSMA) Produce Safety Rule does not demand the addition of sanitizer to produce wash water but requires that if they are in use they must be monitored to ensure adequate treatment and safety (8). A survey conducted among Virginian produce growers in 2017 demonstrates the importance of deepening our knowledge with respect to the use of these sanitizers. Twenty-seven percent of respondents who indicated that they were using a wash system said they did not add sanitizer to their water, and growers that were adding sanitizers to their water were using chlorine, peroxyacetic acid, or both (9). Meanwhile, only 55% of the respondents who were using sanitizer were using the concentration recommended by the manufacturer, while responses ranged from 4 to 7 ppm used in some spray bar wash systems to as high as 200 ppm used in some dump tank-based systems (9). In the same survey, 44% of growers indicated that cost was the most important characteristic when selecting a sanitizer (9).

As for irrigation water, if a water source is found to be unsafe or otherwise inadequate for the intended use, then the FSMA Produce Safety Rule demands that usage stops and corrective actions are implemented before the water may be used again (8). Cl is widely applied within the industry under these circumstances thanks to its availability, ease of use, and efficiency, however, there are growing public health concerns stemming from the fear of hyperchlorination (10). Cl has also been applied to irrigation systems as a means to combat biofilms and reduce the buildup of algae which may clog a system (11), but there is a lack of information on the concentrations at which Cl is being applied, or if an alternative sanitizer, such as PAA, may be better suited for those purposes.

In the United States, foodborne illness from *Salmonella* and shigatoxigenic or pathogenic *E. coli* are responsible for a combined average of over 500 deaths per year, additionally, they are estimated to cost at least $3.7 billion and $400 million per year, respectively (12). Contaminated agricultural water can serve as a vector for these pathogenic microorganisms to make their way into the food supply (13). *Salmonella* contributed 53.4% of all foodborne disease outbreaks from 2006 to 2017, approximately 33% of these *Salmonella* outbreaks were associated with the consumption of produce (13). Trace-back investigations suggest that irrigation water may be a source of *Salmonella* contamination in produce and a vehicle for transmission (13). *E. coli* is a microorganism that is commonly shed in the feces of livestock. Under the circumstances of adjacent land-use, this *E. coli*-containing fecal matter has the potential to runoff and contaminate nearby groundwater which then may be used for irrigation (14), ultimately running the risk of adding harmful bacteria to the food supply and causing an outbreak of foodborne illness.

Protozoan parasites, such as *C. parvum* and *C. cayetenensis*, remain a challenge when it comes to food safety, providing unique difficulties in terms of detection and inactivation, while also being highly infectious and challenging to work within a laboratory setting. In the United States *Cryptosporidium* is a leading cause of illness outbreaks linked to recreational water and water used for food preparation. From 2009 to 2020 there were 9,522 cases of cryptosporidiosis, 333 hospitalizations, and 2 deaths, with the number of infections increasing at the alarming rate of 13% per year (15). *C. cayetenensis* is a particularly challenging organism, currently believed to only infect humans there is no culture method through which the organism’s infectivity can be assessed, meaning a surrogate organism, such as *Eimeria tenella*, must be used (16). That being said, the importance of *C. cayetenensis* as a foodborne pathogen is exploding thanks to an increasing rate of infection. Between 1998 and 2017 there were an estimated 1,823 illnesses from *C. cayetenensis*, followed by 2,173 cases from a single outbreak in July of 2018, and most recently a total of 2,272 *C*. *cayetenensis* infections in 2023 (17), these outbreaks are most frequently attributed to leafy green produce most likely contaminated through agricultural water (18).

A 2020 multistate outbreak, attributed to leafy greens and traced back to a South Florida canal, resulted in the FDA issuing a report on *C. cayetenensis*, urging growers in the area to especially consider the organism when assessing risk and to implement risk mitigation measures where appropriate (19). An objective of this research is to fill in critical knowledge gaps that exist regarding the use of sanitizers in agricultural water to aid growers in this regard, particularly with respect to challenging organisms like protozoan parasites. Furthermore, deepening our understanding of the effective use of these sanitizers will also allow for their more fiscal application, thus improving growing practices in ways that extend even beyond public health.

## RESULTS

**Fig 1:**
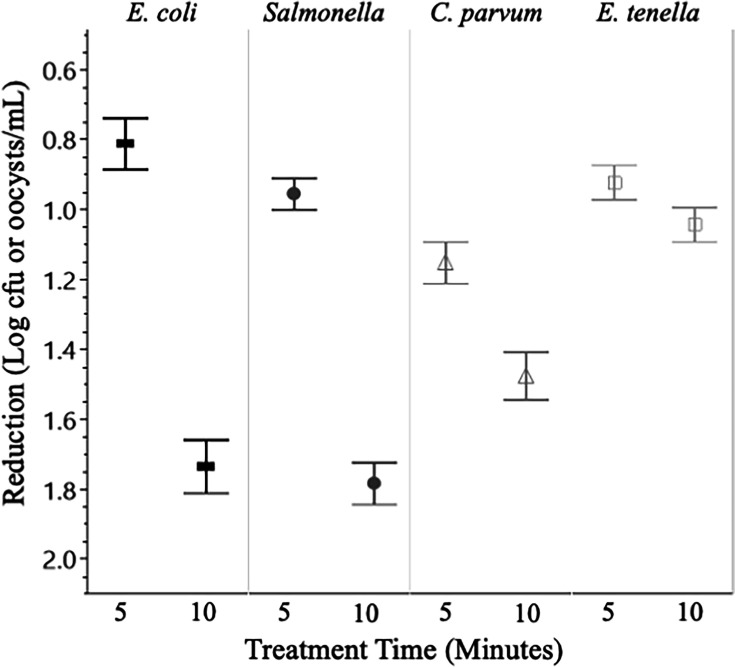
Log reductions with respect to treatment time. Across all experiments, a longer treatment time resulted in significantly (*P* < 0.05) greater organism inactivation. Data presented are the combined averages from experiments conducted in W1 and W2, at 12°C and 32°C, and treatment with all levels of Cl and PAA (3 and 10 ppm for bacteria, 3, 10, 50, 100, and 200 ppm for protozoa). *n* = 108 for bacterial targets, *n* = 112 for protozoa. Error bars are constructed using 1 standard deviation from the mean. ▀ = *E. coli*, *● = Salmonella*, Δ = *C. parvum*, □ = *E. tenella.*

**Fig 2:**
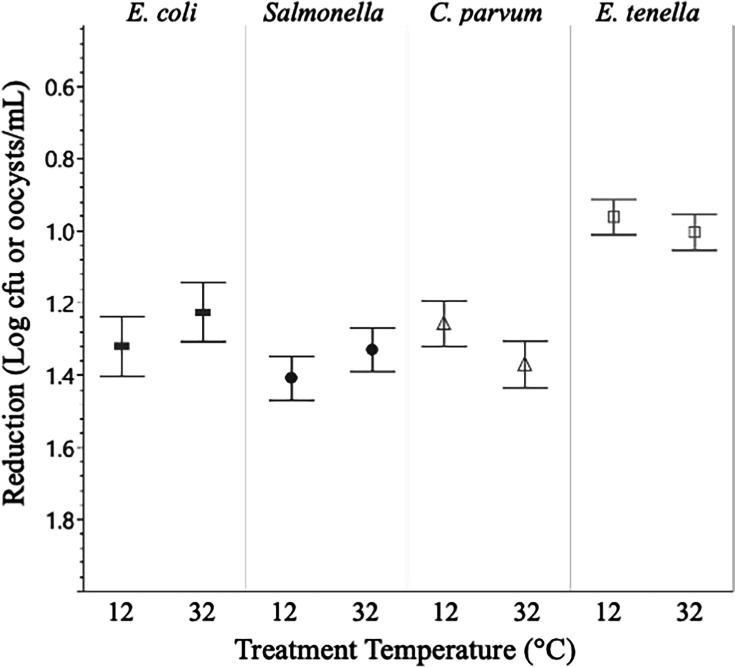
Log reductions by an organism with respect to treatment temperature. Differences in organism recovery between 12°C and 32°C were not significantly (*P* > 0.05) different for any organism. Data presented are the combined averages from experiments conducted in W1 and W2, for 5 and 10 minutes of treatment, and treatment with all levels of Cl and PAA (3 and 10 ppm for bacteria, 3, 10, 50, 100, and 200 ppm for protozoa). *n* = 108 for bacterial targets, *n* = 112 for protozoa. Error bars are constructed using 1 standard deviation from the mean. ▀ = *E. coli*, *● = Salmonella*, Δ = *C. parvum*, □ = *E. tenella.*

**Fig 3:**
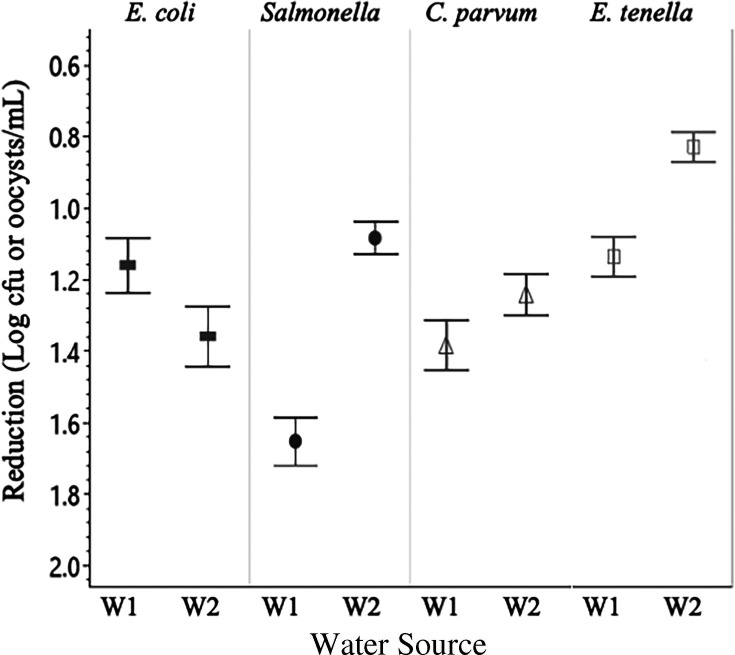
Log reductions with respect to water source. Differences in organism recovery between water sources 1 and 2 (**W1 and W2**) were significant (*P* < 0.05) for all organisms. Data presented are the combined averages from experiments conducted at 12°C and 32°C, for 5 and 10 minutes of treatment, and with all levels of Cl and PAA (3 and 10 ppm for bacteria, 3, 10, 50, 100, and 200 ppm for protozoa). *n* = 108 for bacterial targets, *n* = 112 for protozoa. Error bars are constructed using 1 standard deviation from the mean. ▀ = *E. coli*, *● = Salmonella*, Δ = *C. parvum*, □ = *E. tenella.*

Universally, higher concentrations of sanitizer, as well as longer treatment times (Fig. 1), resulted in greater reductions in organism populations. Conversely, treatment temperature did not result in significant differences between treatments with respect to sanitizers and organisms (Fig. 2). Additionally, there were significant differences in bacterial and parasitic recovery rates between both water sources (Fig. 3), with water source 1 consistently having significantly (*P* < 0.05) higher inactivation for all organisms. Across all experiments, a contact time of 5 minutes with a low concentration of either sanitizer was ineffective at eliminating bacteria and inactivating oocysts. The greatest reductions under these conditions came from *Salmonella* in water source 1 at 32°C, being exposed to a low concentration PAA for 5 minutes, resulting in a 0.75 log reduction.

### Water metrics results

Water sources 1 and 2 were significantly different across all key parameters that were measured (Table 1). Water source 1 (W1) had a pH of 7.7, conductivity of 472 µs/cm, 11.9 mg/L of dissolved oxygen, 511 mg/L total dissolved solids, and a turbidity of 22.5 NTU. Meanwhile, water source 2 (W2) was measured to have a pH of 6.58, conductivity of 192 µs/cm, 32 mg/L of dissolved oxygen, 209 mg/L total dissolved solids, and a turbidity of 31.25 NTU. The results listed are averages of triplicate measurements for each parameter.

**TABLE 1:** Water parameters by source^*a*^

	pH	Conductivity (μS/cm)	Dissolved oxygen (mg/L)	Turbidity (NTU)	Total dissolved solids (mg/L)	Colilert *E. coli* MPN/100 mL
Water source 1 (W1)	7.70* (±0.1)	472* (±35.1)	11.9* (±3.6)	22.5* (±2.7)	511* (±10.6)	57.8 (±4.9)
Water source 2 (W2)	6.58* (±0.16)	192* (±26.9)	32* (±7.6)	31.3* (±5.4)	209* (±33)	39.2 (±2.8)

^*a*^ Numbers displayed are the averages of triplicate readings taken at the time of collection. *, significant (*P* < 0.05) differences between sources.

### Analysis with respect to water metrics

For *E. coli* experimentation, the starting inoculum was determined to be 9.29 log cfu/mL. In the preliminary experiments, when no sanitizer was present, *E. coli* was recovered at a concentration of 9.09 log cfu/mL in W1 and a significantly (*P* < 0.05) lower 8.51 log cfu/mL in W2. This result indicates that W1 was not inhibitory toward *E. coli*, but W2, with an approximately 0.7 log reduction appears to have had some slight inhibitory effects on organism recovery. Notably, *Salmonella* did not share this characteristic, having a starting inoculum of 8.39 log cfu/mL and recovery of 8.36 and 8.15 log cfu/mL from W1 and W2 which was not a significant reduction.

Regarding the parasite targets, across all experiments, 6 logs of oocysts were used as the starting inoculum. It was determined that 94%–99% of oocysts remained infectious after being put through the process with no sanitizer present, suggesting that the water sources provided insignificant reductions in infectivity toward both *C. parvum* and *E. tenella*. Results from these analyses served as non-sanitizer positive controls against which reductions in treated oocyst infectivity were compared.

### Bacterial reduction*—E. coli*

**Fig 4:**
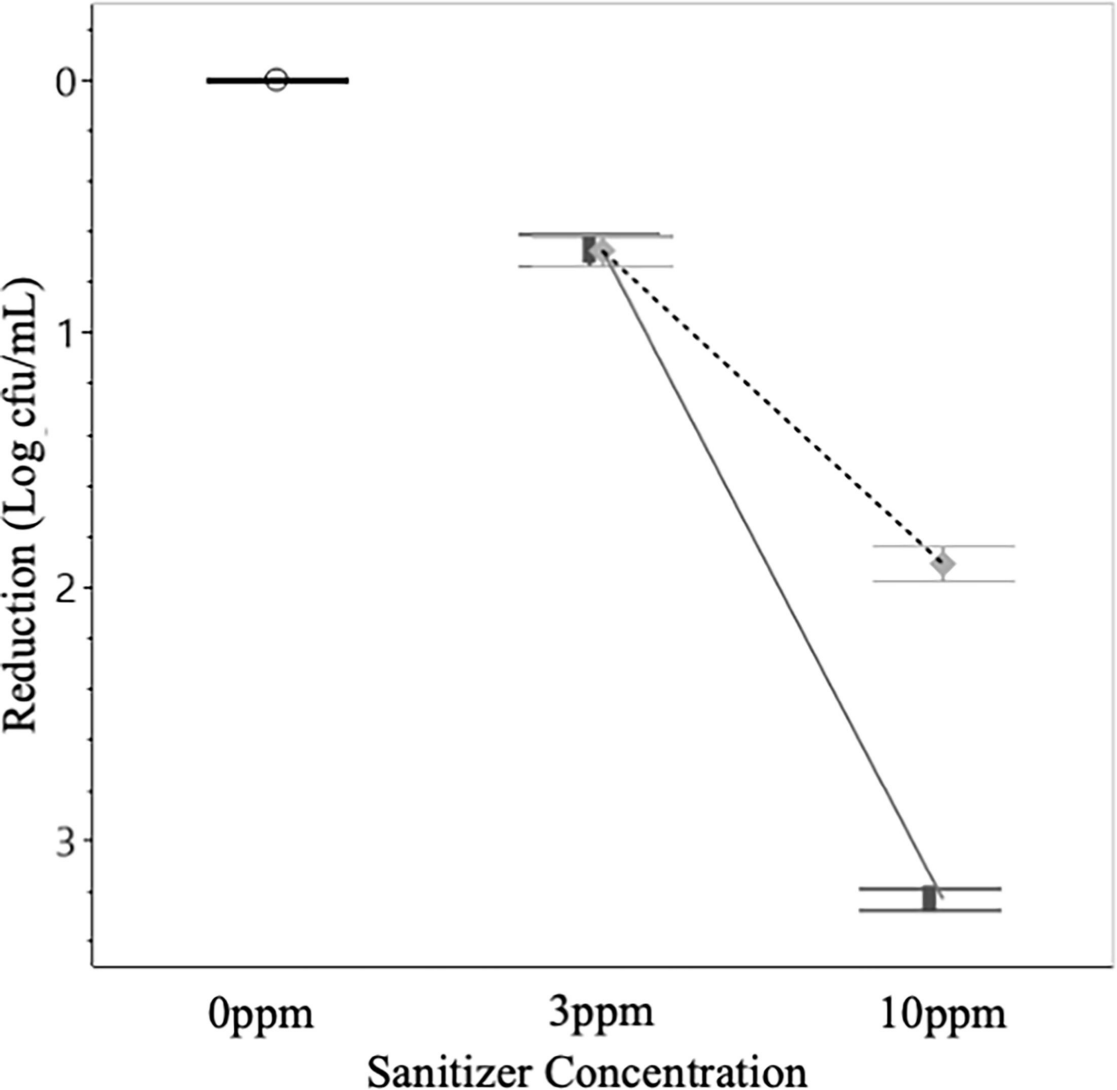
Log reductions in *E. coli* post-sanitizer treatment**.** *E. coli* demonstrated significantly (*P* < 0.05) greater reductions after treatment with Cl as opposed to PAA. Data presented are the combined averages from experiments conducted in W1 and W2 and at 12°C and 32°C after 10 minutes of treatment (*n* = 24 per treatment). Error bars are constructed using 1 standard deviation from the mean. ○ = 0 ppm control, solid line and █ = Cl, dotted line and ♦ = PAA.

Given 10 minutes of contact, low concentrations of sanitizer (3 ppm) were effective at eliminating *E. coli*, resulting in average reductions of 0.83 and 0.89 log cfu/mL for Cl and PAA, respectively. At the higher concentration Cl outperformed PAA substantially, resulting in an average reduction of 3.48 log cfu/mL as opposed to a 2.50 log cfu/mL reduction (Fig. 4). Notably, the single greatest reduction in *E. coli* recovery was found when W1 was treated with 10 ppm Cl, the result of this experiment was a complete elimination of *E. coli*, suggesting an 8 log cfu/mL reduction. Given the dramatic reduction, this set of experiments was replicated 12 times with the bacteria never being successfully recovered, however, this data has been excluded from higher-order data analyses (e.g., the overall effects of Temperature, Time, and Water Source), being treated as an outlier.

### Bacterial reduction*—Salmonella*

**Fig 5:**
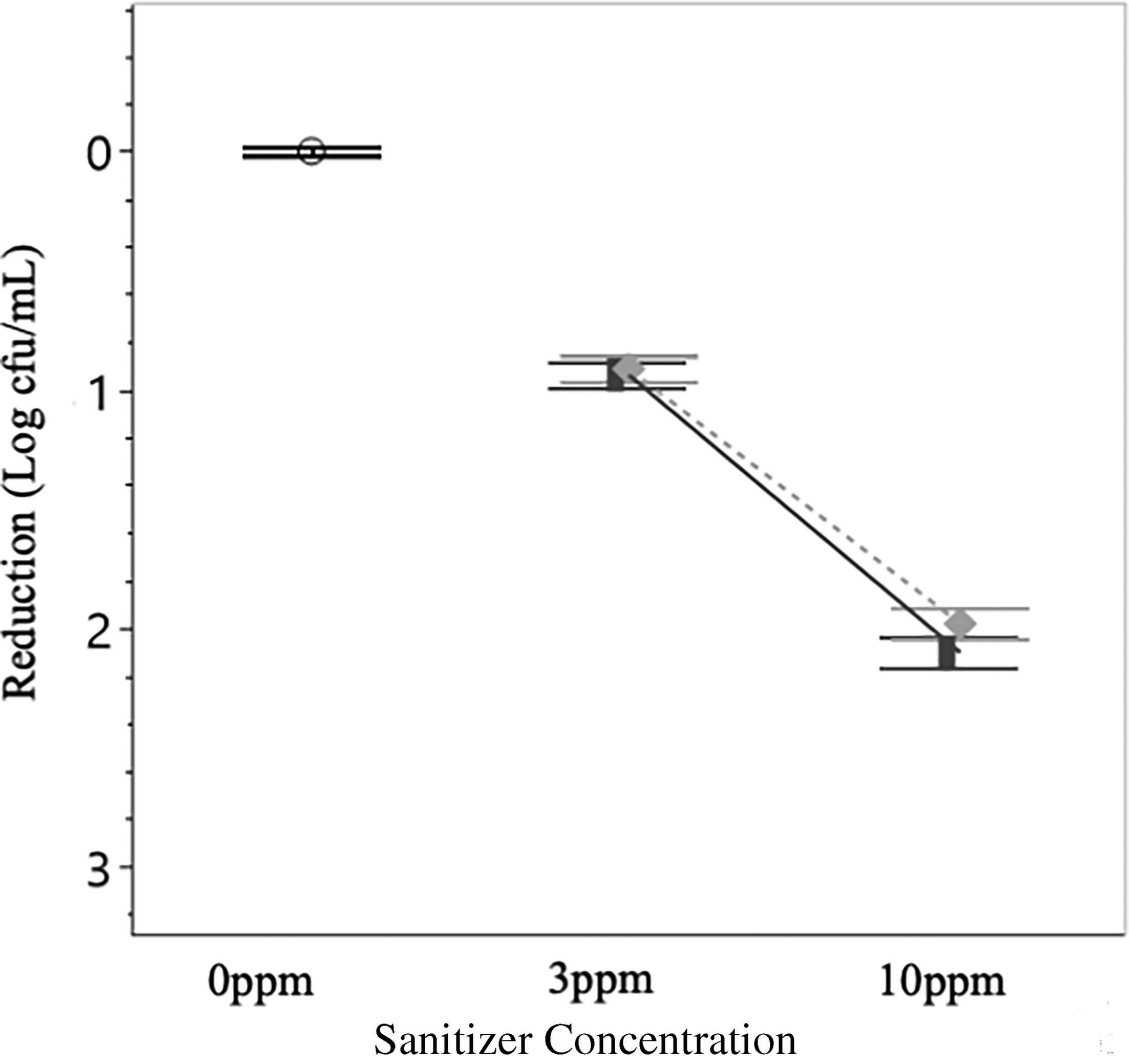
Log reductions in *Salmonella* post-sanitizer treatment. *Salmonella* demonstrated significant (*P* < 0.05) reductions after treatment when exposed to either sanitizer. Differences between Cl and PAA reductions became significant (*P* < 0.05) when sanitizers were applied at higher concentrations. Data presented are the combined averages from experiments conducted in W1 and W2 and at 12°C and 32°C after 10 minutes of treatment (*n* = 24 per treatment). Error bars are constructed using 1 standard deviation from the mean. ○ = 0 ppm control, solid line and █ = Cl, dotted line and ♦ = PAA.

Low concentrations of sanitizer (3 ppm) were effective at eliminating *Salmonella* with neither Cl nor PAA being significantly (*P* > 0.05) more effective, each sanitizer yielded an approximate 1.37 log cfu/mL reduction after 10 minutes. When applied at a concentration of 10 ppm the sanitizers were significantly (*P* < 0.05) more effective, albeit indistinct from one another, resulting in a 2.5 log cfu/mL reduction for both Cl and PAA (Fig. 5).

### Oocyst inactivation*—C. parvum*

**Fig 6:**
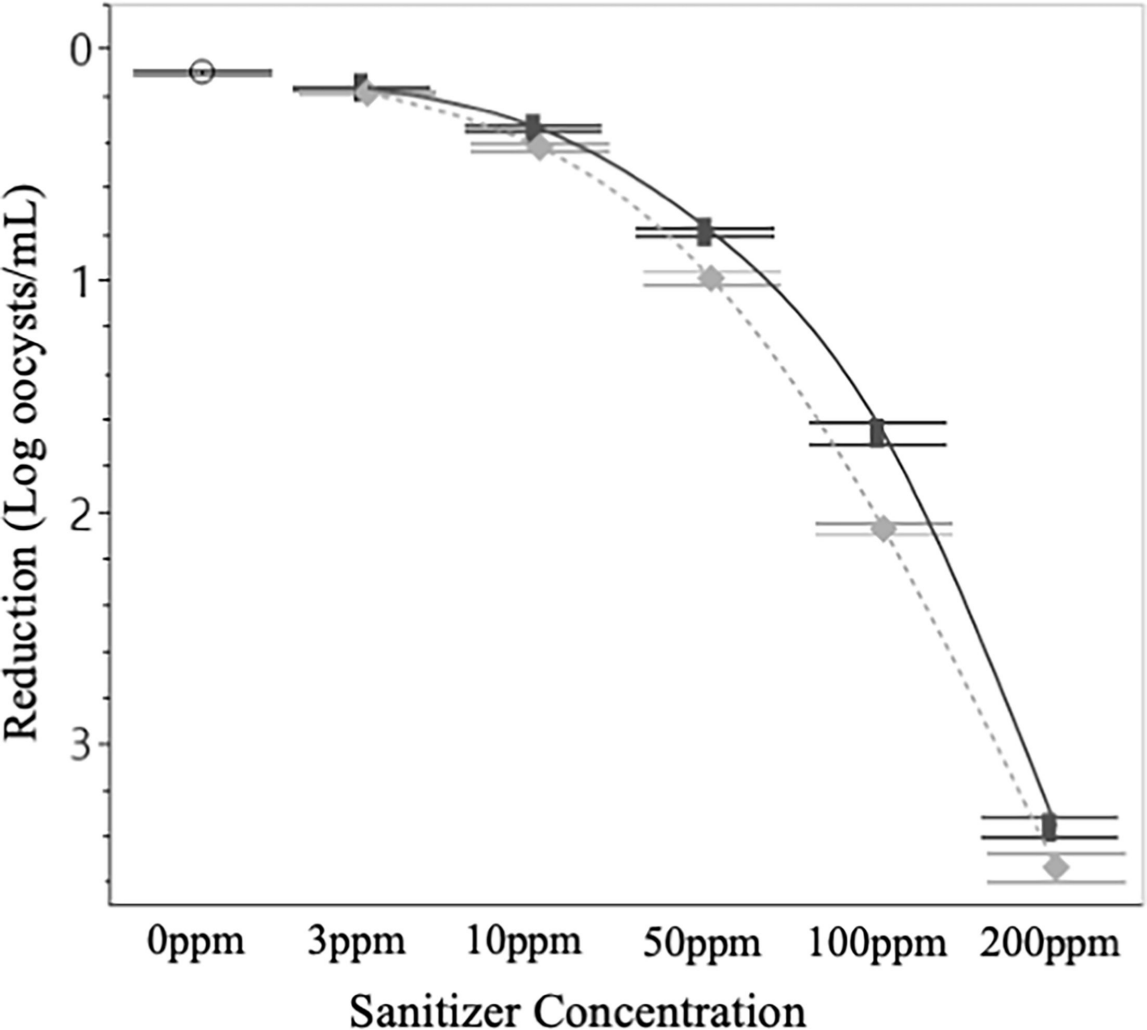
Log reductions in infectious *C. parvum* post-sanitizer treatment. Regardless of sanitizer concentration, oocyst infectivity was significantly (*P* < 0.05) lower after treatment with PAA as opposed to Cl. Data presented are the combined averages from experiments conducted in W1 and W2 and at 12°C and 32°C after 10 minutes of treatment (*n* = 24 per treatment). Error bars are constructed using 1 standard deviation from the mean. ○ = 0 ppm control, solid line and █ = Cl, dotted line and ♦ = PAA.

After 10 minutes of treatment with a low concentration of either sanitizer *C. parvum* oocysts remained fully infectious, showing insignificant reductions in viability. Treatment with 10 ppm of sanitizer resulted in a small yet significant (*P* < 0.05) reduction in oocyst infectivity (0.44 log and 0.54 log reductions for Cl and PAA, respectively). Predictably, even greater reductions in infectivity were observed when sanitizer concentrations were increased (Fig. 6). At 50 ppm, the effects of the Cl and PAA became significantly different from each other, though both remained effective. A concentration of 50 ppm Cl resulted in a 0.94 log reduction in oocyst viability, while PAA achieved a 1.23 log reduction. The greatest reduction (3.8 log) occurred when oocysts were treated with 200 ppm PAA, whereas treatment with 200 ppm Cl led to a 3.61 log reduction. The trend of PAA being significantly (*P* < 0.05) more effective at inactivating *C. parvum* oocysts was consistent at all sanitizer concentrations above 10 ppm.

### Oocyst inactivation*—E. tenella*

**Fig 7:**
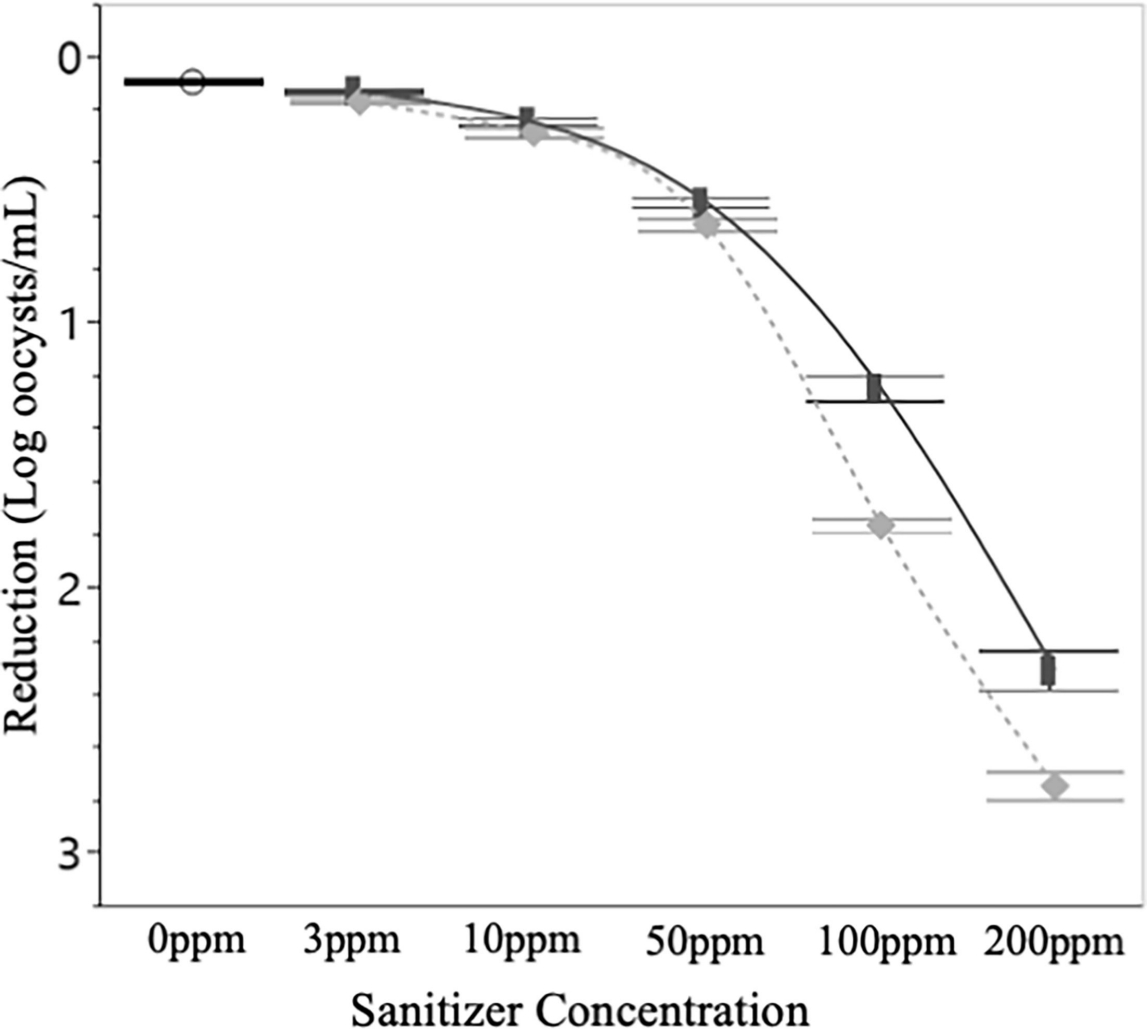
Log reductions in infectious *E. tenella* post-sanitizer treatment. Regardless of sanitizer concentration, oocyst infectivity was significantly (*P* < 0.05) lower after treatment with PAA as opposed to Cl. Data presented are the combined averages from experiments conducted in W1 and W2 and at 12°C and 32°C after 10 minutes of treatment (*n* = 24 per treatment). Error bars are constructed using 1 standard deviation from the mean. ○ = 0 ppm control, solid line and █ = Cl, dotted line and ♦ = PAA.

Treating *E. tenella* oocysts with 3 or 10 ppm of sanitizer was ineffective at decreasing infectivity regardless of 5 or 10-minute contact time (Fig. 7). The greatest reduction under these circumstances came from 10 minutes of treatment with 10 ppm PAA, which resulted in an insignificant (*P* > 0.05) 0.34 log reduction. When sanitizer concentrations were increased to 50 ppm the reductions became significant (*P* < 0.05), with PAA producing a 0.76 log reduction in infectious oocysts and Cl producing a 0.63 log reduction. The discrepancy in the efficacy of these sanitizers became apparent when applied at a concentration of 100 ppm; PAA yielding a 1.87 log reduction and Cl yielding a significantly (*P* < 0.05) lower reduction of 1.37 log. In general treatment with PAA was more effective at inactivating *E. tenella* oocysts as compared to Cl, but this difference was not always significant. Interestingly, the differences between sanitizers were less pronounced at even higher concentrations, with 200 ppm PAA remaining more effective than Cl of equal concentration (providing a 2.61 log reduction as opposed to 2.51) but the difference between them had a comparatively low significance (*P* < 0.05).

## DISCUSSION

The important and pervasive organisms *E. coli* and *Salmonella* are responsible for millions of illnesses each year while also costing producers and the federal government billions of dollars. These pathogens have myriad ways to enter into and disrupt the food supply, some of these ways include being introduced through agricultural water via the necessary irrigation of produce and the washing process, something done as a critical control point to reduce the presence of soilborne microbes (20). The treatment of water with chemical sanitizers is a proven means by which one can reduce these bacterial populations and limit their potential for introduction into the food supply. Chlorine and peracetic acid are both highly effective at eliminating these microbes, with Cl being a bit more efficacious for both species when other variables (water temperature, contact time, etc.) are controlled. The drawbacks of chlorinating agricultural water before application include the fact that Cl tends to carry a negative connotation when it comes to food safety, potentially off-putting consumers, it also tends to remain in the environment much longer than a sanitizer such as PAA (20).

Other studies, using a similar test system and also aiming to compare the effectiveness of Cl and PAA sanitizers to eliminate *E. coli* and *Salmonella* in agricultural water, have been conducted recently. The rates of bacterial inactivation were generally lower in this study (the highest being a 3.48 log reduction of *E. coli* when treated with 10 ppm Cl as opposed to a 7.05 log reduction observed in another study [21]), however, the results here are consistent with their determination that, broadly speaking, Cl is more effective than PAA at eliminating *E. coli* in agricultural water (21). These studies are also consistent in their findings in that higher concentrations of sanitizer, as well as extended treatment times, consistently yielded greater reductions in bacterial inactivation (22).

Protozoan parasites present a number of unique challenges in terms of produce safety, these challenges include the organisms being highly durable in general, as well as capable of surviving in an environment for years while remaining infectious (23). Beyond that, these organisms can be exceptionally difficult to detect and study in a laboratory setting, often requiring unique equipment and specializations (24). However, though these organisms are resilient, they were susceptible to inactivation through the use of either Cl or PAA. Regardless of testing conditions, both *C. parvum* and *E. tenella* (acting as a surrogate for *C. cayetenensis*) in agricultural water were able to be reliably inactivated when treated with Cl or PAA, with PAA being significantly more effective than Cl when used at high concentrations (>50 ppm). The benefits of PAA over Cl, with regard to the control of protozoan parasites, were consistent across all experiments, suggesting that PAA should likely be the sanitizer of choice for the control of protozoan parasites which may contaminate produce via water, such as *C. cayetenensis*, which we are yet to fully understand but are becoming ever more prevalent.

Notably, and unlike the bacterial targets, *C. parvum* and *E. tenella* had significantly (*P* < 0.05) different sensitivities to sanitizer treatment at all levels greater than 10 ppm, with *C. parvum* always being more easily inactivated. One possible explanation for this vulnerability may be that *C. parvum* oocysts have a suture- or seam-like structure (25), this structure serves as an exit for the sporozoites during excystation but may also be acted upon or penetrated by the sanitizers during treatment. *E. tenella* oocysts, on the other hand, rely on several surface proteins, which detect the immune response of the host organism, to signal favorable conditions and trigger excystation (26). *C. parvum* and *E. tenella* also differ in their internal structures, unlike *C. parvum*, *E. tenella* oocysts (as well as those of *C. cayetenensis*) contain subunits called sporocysts which house the infectious sporozoites (16), and may offer them another layer of protection should the oocyst itself be penetrated. In short, *E. tenella* oocysts appear to require a more nuanced set of circumstances before excysting, and have additional layers of protection for their sporozoites, suggesting a more complex and robust oocyst than that of *C. parvum*, granting them enhanced resistance to chemical sanitizer treatment which act upon the oocyst.

The differences between sanitizers and their interactions with different microbes can be dramatic, with hardier organisms, such as protozoan parasites, being demonstrably less susceptible to sanitation than bacteria in agricultural water matrices. The tough oocysts encapsulating the infectious units of protozoan parasites allow them to escape the effects of sanitizers^2^, unlike in the bacteria, which are lacking in any such protection and are readily eliminated thanks to the chemical activity of sanitizers such as PAA and Cl. It is worth noting that the studied protozoan parasites required treatment with more than ten times the concentration of sanitizer to see reductions similar to those observed in bacteria. Interestingly, bacteria appear to demonstrate greater susceptibility to treatment with Cl as opposed to PAA, while parasitic organisms were marginally more vulnerable to treatment with PAA.

In the context of environmental persistence, both protozoan parasites and bacteria have demonstrated the ability to remain infectious and problematic for extended periods. In agricultural soil, *E. coli* O157:H7 can persist for a period of up to 196 days (27) and *Salmonella* can persist for over 200 days depending on soil conditions (28). Data for the persistence of protozoan parasites is more limited, however, it has been estimated that *C. parvum* oocysts would remain infectious for approximately 160 days in field soil while temperatures fluctuated between 0°C and 1°C (29). As for *E. tenella*, recent research has shown that oocysts stored in sporulation media at 12°C remain viable for 10 months (29), and much earlier studies suggest that the oocysts can survive in soil for over 600 days (30). While there is a consensus that these organisms have the potential to survive very long periods in agricultural matrices (such as soil or plant matter), there is a need for research evaluating the decay of these organisms in environmental conditions.

Overall, when selecting a sanitizer to treat one’s agricultural water a number of factors are to be considered to make a pragmatic decision. Cl is the cheaper option, having a long history of use in food production there are a number of suppliers, and it is readily available (31). However, if one wishes to control protozoan parasites more effectively, PAA is the superior sanitizer, while also boasting the ability to dissipate harmlessly into the environment after application (32). It is important to note that treatment time was a major factor in sanitizer efficacy, when comparing 5-minute to 10-minute treatments the longer treatment time consistently produced reductions in microbe populations that were similar to using the sanitizers at a much higher concentration (i.e., 10 minutes of contact with a 3 ppm sanitizer had results similar to 5 minutes of contact with a 10 ppm sanitizer).

It is difficult to ascertain precisely why water source 1 had consistently lower organism recovery across the suite of experiments. This is due to the fact that these environmental waters had several key characteristics, all of which contribute to the effectiveness of sanitizers, that were significantly different from one another. The properties of each water source can play unique roles when it comes to the chemical activity of the sanitizers as well as the water source’s capacity to support a microbial population in general (4). As such, the isolation and manipulation of these properties individually would allow for a much deeper understanding of what type of environment these microbes are able to thrive in and which properties are most important to facilitate a strong chemical action out of the applied sanitizer. That being said, a more diverse selection of water sources, or replication of these experiments in more unique water sources, would also contribute to this research and allow for a better understanding of which sanitizer will work best under more eclectic conditions.

While the application of a 200 ppm sanitizer is likely prohibitive to most producers, PAA appears to be the most forward-thinking option for sanitizer. Given that protozoan parasites are tougher than bacteria in general, they must be specially considered when deciding on which sanitizer to use, and the results demonstrate that PAA is markedly more effective at the control of these organisms, while also being highly effective in the elimination of bacterial pathogens. In terms of balancing practicality and affordability, even though it is the more expensive option, the use of a medium concentration of PAA (50 ppm or greater) for a longer period (10 minutes) would likely result in greater efficiency and prove to be highly effective at the control of most harmful pathogens present in agricultural water, while also leaving little-to-no residue or runoff into the environment.

## MATERIALS AND METHODS

### Water collection and analysis

Forty liters of water were collected in carboys from two separate agricultural ground-water sources (W1 and W2) in the Mid-Atlantic United States with key parameters measured, in triplicate, at the time of collection. Water was stored at 4°C for 12 weeks, during which time subsamples were periodically taken for use in experiments. All experiments were conducted in both water sources, and the resident microbiota of the water was left unaltered over the course of the experiments. Key parameters included water temperature, air temperature, pH, conductivity, dissolved oxygen, and turbidity. Additionally, 100 mL of each water sample was used in an IDEXX Colilert Quanti-Tray to determine the presence of *E. coli* and enumerate coliforms. The materials used to measure each parameter are outlined in Table 2.

**TABLE 2:** Materials used to measure water parameters

Equipment name	Metric	Manufacturer/ID
pH probe	pH	VIVOSUN pH Meter
TDS meter	Total dissolved solids conductivity	VIVOSUN TDS&EC Meter
Dissolved oxygen analyzer	Dissolved oxygen	RCYAGO DO9100
Turbidity test kit	Turbidity	LaMotte 7519-01
Quanti-Tray 2000	MPN *E. coli* and coliforms	IDEXX
PAA test strips	Low PAA concentration High PAA concentration	BartOvation PPA01V100 BartOvation PPA04V50
Chlorine test strips	Low Cl concentration High Cl concentration	BartOvation PCL02V50 BartOvation PCL05V100

^*a*^ Measurements taken in triplicate at the time of water collection.

### Organism acquisition and preparation

The seven strains of *Salmonella* and *E. coli* outlined in the EPA Agricultural Water panel (33) were generously gifted by the University of Arizona, Department of Environmental Sciences, and acclimatized to be Rifampicin prior to receipt. The *E. coli* used were serotypes O157:H7 (ATCC 43895), O26:H11 (ATCC BAA-2196), O45:H2 (ATCC BAA-2193), O103:H11 (ATCC BAA-2215), O111 (ATCC BAA-2440), O121:H19 (ATCC BAA-2219), and O145:Nonmotile (ATCC BAA-2192), and the *Salmonella* used were *Salmonella enterica* subsp. *enterica* strains Cerro (ATCC BAA-3136), Give (ATCC BAA-3137), Newport (ATCC BAA-3138), Poona (ATCC BAA-3139), Rubislaw (ATCC BAA-3140), Thompson (ATCC BAA-3141), and Typhimurium (ATCC BAA-3142). Strains were individually grown from frozen bacterial stocks by streaking onto Tryptic Soy Agar plates which were then incubated at 37°C for 24 hours. The following day single colony isolates were transferred into 15 mL conical centrifuge tubes containing 10 mL of Tryptic Soy Broth and again incubated for 24 hours at 37°C. Following incubation, in duplicate, 1 mL was taken from each broth culture and combined in 15 mL conical centrifuge tubes which were then centrifuged at 8,000 × *g* for 3 minutes, the supernatant was then decanted, and the pellet resuspended in 1 mL of Buffered Peptone Water (BPW). Cultures were enumerated on MacConkey agar for the isolation of *E. coli* (34) or XLT4 agar (for the isolation of *Salmonella*) (34) both supplemented with 80 ug/mL Rifampicin (35).

*C. parvum* oocysts (Iowa isolate) were obtained from Waterborne Inc. (New Orleans, LA), where they were propagated in experimentally infected calves and stored in PBS with 0.01% Tween 20, supplemented with penicillin, streptomycin, gentamicin, and amphotericin B. The viable oocysts were stored at 4°C before use in experimentation. *E. tenella* oocysts were generously provided by the USDA-ARS Animal Parasitic Diseases Laboratory (Beltsville, MD). *E. tenella* oocysts are propagated in experimentally infected poultry and collected from the ceca. Oocysts were then washed and stored in potassium dichromate at 4°C before use in experiments within 6 months of collection. *E. tenella* was used as a surrogate for *C. cayetenensis* in these experiments as they share several biological characteristics and have closely related genetics (36). It is also worth noting that there are biological discrepancies between the organisms; for example, sporulation is more rapid in *Eimeria* spp., ranging from 24 to 48 hours as opposed to *C. cayetenensis’* 7 to 15-day period, and the organisms differ in how many sporocysts are present within their sporulated oocysts, *Eimeria* spp. containing four and *C. cayetenensis* containing two (16). These traits may offer increased or decreased resistance to chemical treatment, thus complicating the task of predicting how *C. cayetenensis* may behave under the same circumstances.

### Test matrix and sanitizer preparation

One molar solution of Cl and PAA was prepared in deionized water using Accu-Tab calcium hypochlorite tablets and Biosafe Systems SaniDate12.0, respectively. These stocks were then serially diluted 1:10 in deionized water to generate a stock 1,000 ppm solution. Dilutions of each sanitizer were tested using Free Chlorine and Peracetic Acid test strips, respectively, until a 1,000 ppm solution was achieved. The 1,000 ppm solution was then further diluted into aliquots of the collected Ag water to generate Sanitizer/Ag. Water solutions of 4–6, 10–12, 50, 100, and 200 ppm, the concentrations of these working stocks were verified using low-range Free Chlorine and Peracetic Acid test strips immediately before inoculation with the target organism (37).

### Treatment of organisms

*E. coli* and *Salmonella* inactivation were evaluated using the bacterial cocktails and Sanitizer/Ag. In water solutions described above, bacteria were exposed to Cl and PAA at concentrations of 3 and 10 ppm. In an incubator, triplicate 98 mL aliquots of Sanitizer/Ag. Water solutions were brought to the desired experimental temperature (12°C or 32°C) and 1 mL of the bacterial cocktail was added. After 5 or 10 minutes of contact, 1 mL of 1 M sodium metabisulfite solution was added to the mixture to neutralize the activity of the sanitizer, complete neutralization was confirmed via test strip. The treated samples were then serially diluted in BPW and the surviving cultivable bacteria were enumerated on MacConkey or XLT4 agar supplemented with 80 ug/mL Rifampicin.

*C. parvum* and *E. tenella* oocyst experiments were also conducted using the Sanitizer/Ag. Water solutions. Aliquots of the Sanitizer/Ag. Water solutions were inoculated with 10^6^ oocysts, of which greater than 95% were sporulated, and the solutions contained the same “low” levels of sanitizer used in bacterial experiments (3 and 10 ppm) as well as “high” concentrations of each sanitizer (50, 100, and 200 ppm). Experiments were conducted in triplicate and repeated at 12°C and 32°C, and contact times of 5 and 10 minutes were also evaluated. After the desired contact time and sanitizer neutralization, the samples were centrifuged at 2,000 × *g* for 20 minutes, the supernatant was aspirated and the pellet was resuspended in 1 mL of PBS. Centrifugation and resuspension in PBS were repeated three times to rinse the oocysts in preparation for addition to mammalian cell culture to assess infectivity.

### Cell culture maintenance, infectivity assays, and molecular detection

*C. parvum* infectivity was assessed in human ileocecal adenocarcinoma cells (HCT-8 Cells, ATCC CC-244, American Type Culture Collection, Manassas, VA) maintained in RPMI-1640 media (Mediatech Inc., Manassas, VA) and managed per (38). After sanitizer treatment, and washing as previously described (38) the oocysts were resuspended in 800 uL of sterile PBS and added to confluent cells in 6-well plates. The plates were incubated at 37°C for 60 minutes to allow infection to occur, after which time the inoculum was removed and 2 mL of maintenance media was added, and the plates incubated for 48 hours. After incubation, the maintenance media was removed and replaced with 0.25% Trypsin, the plates were incubated for 10 minutes and the detached cells were collected for nucleic acid extraction.

To assess *E. tenella* infectivity Madin-Darby Bovine Kidney cells (MDBK cells, ATCC NBL-1, American Type Culture Collection, Manassas, VA) were maintained in DMEM media (Mediatech Inc., Manassas, VA) and managed per (39). After sanitizer treatment and washing per (38), the oocysts were resuspended in 800 uL of sterile Hanks’ balanced salt solution (HBSS) and added to confluent cells in 6-well plates. The plates were then incubated at 37°C for 60 minutes to allow infection to occur, after which time the inoculum was removed and 2 mL of maintenance media added, and the plates incubated for 48 hours. Following incubation, the monolayers were gently washed in triplicate with HBSS to remove unadhered sporozoites. The cells were then trypsinized and returned to the incubator for 10 minutes, after which the unadhered cells were collected for nucleic acid extraction.

Nucleic acid extraction was performed per Table 3, followed by triplicate qPCR amplification. PCR primer and probe sequences are outlined in Table 4 and were performed as described for *C. parvum* (38) and *E. tenella* (40). All qPCR reactions were performed using a Rotor Gene Q Thermocycler (Qiagen, Hilden, Germany) using the following cycling parameters: hold: 95°C for 5 minutes, 40 cycles of denaturation, and combined annealing/extension at 95°C for 5 seconds and 60°C for 10 seconds, signal acquisition during combined annealing/extension.

**TABLE 3:** Materials used for mammalian cell culture assay and nucleic acid extraction^*a*^

	Mammalian cells	Cell culture media	Nucleic acid extraction kit
*C. parvum*	HCT-8 (Human Ileocecal Adenocarcinoma)	Growth: RPMI + 10% FBS Maintenance: RPMI + 2% FBS Detachment: Trypsin 0.25%	Zymo Quick-DNA MiniPrep (Cat: D3025)
*Eimeria tenella*	MDBK (Madin-Darby Bovine Kidney)	Growth: DMEM + 10% FBS Maintenance: DMEM + 2% FBS Detachment: Trypsin 0.25%	Zymo Quick-DNA MiniPrep (Cat: D3025)

^*a*^ Nucleic acid extraction was performed following the user manual provided with each kit through the recommended cell monolayer sample method.

**TABLE 4:** Primers and probes used for the detection of parasite nucleic acid and qPCR reaction mixture and cycling parameters

		Primer/probe sequences (5’ - 3’)	Genomic target
*C. parvum*	Forward	TCC TCT GCC GTA CAG GAT CTC TTA	CPHSPT2F (346 bp target)
	Reverse	TGC TCT TAC CAG TAC TCT TAT CA	
*E. tenella*	Forward	TCA TCA CCC AAA GGG ATT	5s rRNA (278 bp target)
	Reverse	TTC ATA CTG CGT CTA ATG CAC	
	Probe	Fam-CGC CGC TTA ACT TCG GAG TTC AGA TGG GAT-Tamra	

In addition to treated oocysts, HCT-8 and MDBK cells were infected with 10^6^ untreated oocysts. The monolayers were recovered and genetic material was extracted and serially diluted 10-fold, these dilutions were then run alongside the genetic material extracted from the treated oocysts acting as the qPCR standard curve. Performing an infectivity assay followed by qPCR allows for the enumeration of oocysts that remained infectious after exposure to the sanitizers; oocysts that are rendered non-infectious or unable to excyst will be removed through the washing steps and only organisms that successfully invade the cell culture will be collected after trypsinization.

### Evaluation of process toxicity and organism recovery

In addition to the outlined experimental parameters, the survival and recovery potential of each organism was assessed in the absence of sanitizer to ensure the water sources themselves and experimental design were not inhibitory to the organisms. Experiments were conducted in the same manner as those described above without the addition of sanitizer. Organism survival was evaluated 5 and 10 minutes post-inoculation into the water, and experiments were repeated at both 12°C and 32°C. These results were considered as process-control and organism recovery post-treatment was assessed in relation to these results.

### Data analysis

Across the study, experiments were performed in triplicate with bacterial enumeration and molecular detection methods were also conducted in triplicate. Data were analyzed and figures were generated using JMP Pro 16 and Microsoft Excel statistical software. One-way analysis of variance and Tukey HSD were performed to assess the reductions in bacterial populations and inactivation of oocysts for significant differences between toxicity control experiments and sanitizer experiments. *P*-values less than 0.05 were considered statistically significant, with an alpha level of 0.05 (α = 0.05) and a confidence interval of 95%, and error bars reported were constructed using 1 standard error from the mean. In order to evaluate the effects of individual experimental parameters (water source, contact time, and treatment temperature), data presented in Fig. 1 to 3 are the combined averages from experiments conducted using all levels of Cl and PAA as well as the averages from the characteristics not shown in the figure. Data presented in Fig. 4 to 7 are the combined averages of experiments conducted in both water sources and under both treatment temperatures after a contact time of 10 minutes.
